# Effects of Combined Heat and Hypoxic Training on the Physiological Responses During Submaximal Interval Exercise in Untrained Men

**DOI:** 10.3390/jfmk11030344

**Published:** 2026-08-31

**Authors:** Anna Kałuża, Małgorzata Bagińska, Tomasz Pałka, Magdalena Wiecek, Zbigniew Szygula, Marcin Maciejczyk

**Affiliations:** 1 Doctoral School of Physical Culture Science, University of Physical Culture, 31-571 Kraków, Poland; anna.kaluza@doctoral.awf.krakow.pl; 2 Department of Water Sports, Institute of Sport Sciences, University of Physical Culture, 31-571 Kraków, Poland; 3 Department of Physiology and Biochemistry, Institute of Biomedical Sciences, University of Physical Culture, 31-571 Kraków, Poland; tomasz.palka@awf.krakow.pl (T.P.); magdalena.wiecek@awf.krakow.pl (M.W.); 4 Department of Sports Medicine and Human Nutrition, Institute of Biomedical Sciences, University of Physical Culture, 31-571 Kraków, Poland; zbigniew.szygula@awf.krakow.pl

**Keywords:** hypoxic training, heat stress, submaximal exercise, exertion, physiological markers, adaptation, acclimatization

## Abstract

**Background and Objectives**: Aerobic submaximal interval training performed under hypoxic and elevated temperature conditions may induce significant alterations in physiological responses. Understanding these effects may contribute to optimizing training programs for athletes and individuals exposed to extreme environmental conditions. The aim of this study was to investigate the impact of combined heat and hypoxia training on physiological response and adaptation during submaximal interval training in untrained men. **Methods**: Eighty men aged 19–26 years, reporting moderate physical activity levels, were randomly assigned to one of five groups: normoxic thermoneutral training (NOR+21 °C), intermittent hypoxic training in normothermia (IHT+21 °C), normoxic training in heat (NOR+31 °C), intermittent hypoxic training in heat (IHT+31 °C), or a control group (CON). Training sessions were conducted three times per week over a four-week period (12 sessions in total). Somatic parameters and a graded exercise test were assessed before and after the intervention. On the first and last training sessions, selected physiological indices were recorded, including the Bedford scale, Borg Rating of Perceived Exertion (RPE), heart rate (HR), tympanic temperature (Ttym), and skin temperature (Tsk), hematocrit (Hct), and hemoglobin (Hb) and lactate concentration. Statistical analyses included descriptive statistics and analysis of variance (ANOVA) with Tukey’s post hoc test (*p* < 0.05). **Results**: A statistically significant decrease in heart rate (*p* = 0.04) and exercise intensity expressed as a percentage of maximal heart rate (%HRmax) (*p* = 0.03) was observed in the IHT+21 °C group following the training intervention. Furthermore, a significant decrease in RPE was found in the IHT+21 °C (*p* = 0.04) and IHT+31 °C (*p* = 0.001) groups. A significant change in tympanic temperature was noted only in the IHT+21 °C group, where it increased post-intervention (*p* = 0.01). No significant changes in Hct and Hb were observed as a result of training. **Conclusions**: The combination of hypoxic and heat training did not yield the expected results, and no significant changes in the physiological response to submaximal aerobic training were observed in untrained men. In untrained young men, training under hypoxic and thermoneutral conditions leads to significant physiological adaptations, including reductions in heart rate and relative exercise intensity (%HRmax) after the intervention. Moreover, both hypoxia alone and hypoxia combined with heat were associated with a reduced perception of exertion following the training period.

## 1. Introduction

Perceived exertion is defined as the subjective intensity of effort, strain, discomfort, and/or fatigue experienced during physical exercise [1]. The perception of exertion and thermal comfort results from a complex interaction between physiological, environmental, and psychological stimuli [2,3]. Rating scales such as the Borg’s Rating of Perceived Exertion (RPE) [4] and the Bedford thermal comfort scale [5] remain indispensable tools in training practice for assessing the subjective burden of exercise.

Fatigue during demanding physical effort arises due to the depletion of energy reserves, thermoregulatory strain, dehydration, and neurochemical changes [6]. Although exercise intensity plays a key role in the perception of fatigue, it is not the sole factor influencing the subjective assessment of workload. Fatigue is also associated with fluctuating perceptual states and impaired central nervous system functioning, which affect the ability to initiate and sustain effort [7]. Environmental conditions—including ambient temperature, air humidity, and oxygen availability—can intensify physiological responses and amplify the subjective sense of effort [8,9]. Research indicates that perceived exertion under heat stress and hypoxic conditions is significantly higher than under normothermic and normoxic conditions, even at equivalent physical workloads [10], potentially due to increased thermoregulatory and respiratory strain.

During exercise in elevated temperatures, the body must maintain thermal equilibrium, which leads to increases in core temperature (Tc), cardiovascular load (increased HR), profuse sweating, and neurochemical disturbances. These responses collectively impair physical performance [11,12]. Additionally, air humidity is a critical determinant of thermal comfort [13]. Studies show that at relative humidity levels above 70% and ambient temperatures between 30 °C and 32 °C, both the subjective sensation of heat and average skin temperature rise markedly compared to lower humidity environments [13,14]. When heat is combined with high humidity, the primary thermoregulatory mechanism—sweat evaporation from the skin surface—is compromised [15,16]. This reduces thermoregulatory efficiency and exacerbates heat stress. It is hypothesized that fatigue during hyperthermia results from the interplay of peripheral, central (e.g., catecholamine synthesis and activity), and perceptual mechanisms within an integrated physiological system, with elevated brain temperature contributing to central fatigue [12,17,18]. Exercise in hot and humid environments may therefore reduce physiological capacity and voluntary performance by increasing perceived exertion and decreasing thermal comfort, ultimately shortening time to exhaustion [19].

Although hypoxia imposes a substantial burden on the respiratory and cardiovascular systems, it has been suggested that heat-related adaptive mechanisms (e.g., improved blood and oxygen distribution to working muscles) may paradoxically facilitate exercise under hypoxic conditions and attenuate subjective workload perception [20]. In addition to cutaneous vasodilation, elevated temperatures during exercise promote local vasodilation in working muscles, ensuring adequate blood flow [21]. Moreover, the oxyhemoglobin dissociation curve shifts to the right in heat conditions, enhancing oxygen unloading from hemoglobin to muscle tissue [22], potentially reducing the sensation of hypoxia despite lowered oxygen saturation. Exercise in hot environments has been shown to improve muscle deoxygenation (ΔHHb), even without changes in oxygen uptake (VO_2_) [23]. This mechanism, along with other heat-acclimation responses—such as increased plasma volume, improved thermoregulation, reduced cardiovascular strain, and lower RPE [24,25]—may partially compensate for hypoxic stress and reduce perceived workload compared to hypoxia alone.

Heat and hypoxic exposure may induce distinct and potentially opposing responses in blood-volume regulation. Repeated heat exposure is commonly associated with a rapid expansion of plasma volume, which may develop within the first few days of acclimatization and is related, among other factors, to increased sodium and water retention and changes in intravascular protein balance. Because erythrocyte volume generally remains relatively stable during the early stages of heat acclimation, the expansion of the plasma compartment may result in relative hemodilution, reflected by reductions in hemoglobin concentration (Hb) and hematocrit (Hct) [26,27].

In contrast, acute exposure to hypoxia, particularly at altitude-equivalent oxygen levels, may be accompanied by a rapid reduction in plasma volume and consequent hemoconcentration [28]. Under these conditions, increases in Hb and Hct may primarily reflect a reduction in the plasma compartment rather than an immediate increase in erythrocyte volume or total hemoglobin mass. With prolonged hypoxic exposure, however, progressive erythropoietic adaptations may additionally modify red blood cell volume and total hemoglobin mass [29].

Accordingly, when heat and hypoxia are applied separately or simultaneously, the interpretation of Hb and Hct requires consideration of the duration and timing of the exposure, as well as the distinction between changes in blood concentration and changes in circulating cell mass. Monitoring Hb and Hct may therefore provide useful information on the direction and approximate magnitude of hematological responses under different environmental conditions. In the present study, these parameters were also used to estimate exercise-induced changes in plasma volume (ΔPV) using the Dill and Costill equation, as modified by Harrison et al. [30,31].

Despite growing interest in the effects of individual environmental factors, there is a paucity of research examining the cumulative impact of hypoxia and heat on physiological responses, perceived exertion, and adaptive changes. By utilizing laboratory-controlled environmental conditions and a comprehensive assessment of both physiological (heart rate—HR, percentage of maximal heart rate—%HRmax, tympanic temperature—Ttym, skin temperature—Tsk) and subjective measures (RPE and Bedford thermal comfort scale), the present study aims to provide novel insights into the synergistic effects of combined environmental stressors. This approach may enhance the understanding of adaptive mechanisms relevant for optimizing training strategies, particularly for athletes exposed to extreme environmental conditions, such as competitions at high altitudes in hot climates.

It is essential to distinguish between the concepts of adaptation and acclimatization. Adaptation represents a long-term, evolutionary process, whereas acclimatization refers to short-term and reversible physiological adjustments that occur in response to exposure to specific environmental conditions [22,32]. In the present study, we focus exclusively on acclimatization processes induced by exposure to hypoxic and hyperthermic conditions.

The main objective of this study was to determine the effects of training in different environmental conditions on the physiological response during submaximal interval exercise. The detailed objective of the study was to analyze and compare the effects of submaximal interval training performed under four distinct environmental conditions (combined normoxia, hypoxia, 21 °C, and 31 °C) on subjective perceptions of fatigue and thermal comfort, assessed using the Borg and Bedford scales, as well as on selected physiological indicators. In addition, the aim of the study was to assess adaptation to training under varying environmental conditions, in which different environmental stressors were combined. Our study focuses primarily on the impact of heat on physiological response in submaximal interval training, and in particular on assessing the impact of two strong stressors, i.e., heat and hypoxia, as both are important in sports training and are used to maximize exercise capacity.

We hypothesized that the greatest changes in the examined physiological responses to submaximal interval training would be observed under the combined conditions of hypoxia and heat, compared with training performed in hypoxia or heat alone.

## 2. Materials and Methods

### 2.1. Study Design

**Figure 1:**
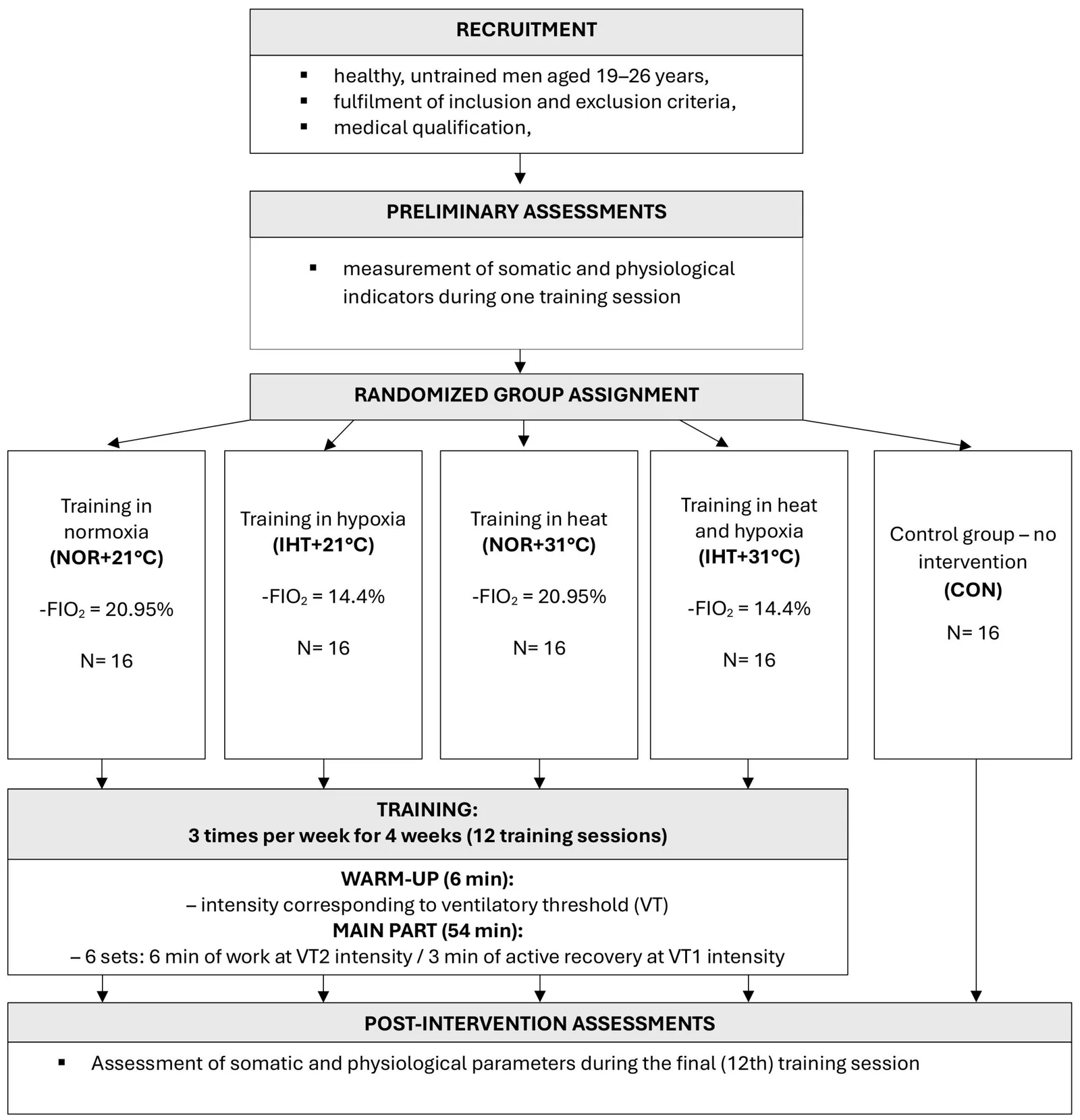
Block Diagram of the Study Design.

The study design comprised several distinct stages, which are outlined below. The study protocol included medical qualification, somatic measurements, and the assessment of selected physiological parameters (Figure 1).

In the first stage, somatic measurements were performed, followed by a graded exercise test used to evaluate the participants’ aerobic capacity and to determine individualized training loads. The main part of the study consisted of training performed under different environmental conditions. After being assigned to specific groups, participants completed 12 training sessions over a 4-week period (three sessions per week). During the first and the final training sessions, changes in selected somatic, physiological, and psychophysiological parameters were monitored. Subsequently, the magnitude and significance of the observed changes between the first and the last training sessions (pre- and post-exercise) were assessed, and the responses to the same exercise bout at the beginning and the end of the intervention were compared.

Eighty male participants aged 19 to 26 years voluntarily enrolled in the study. Prior to the intervention, all individuals underwent a medical screening to rule out health conditions that could preclude participation. This screening included a graded exercise test with electrocardiographic (ECG) monitoring and a complete blood count analysis. All participants were healthy and deemed fit to undergo performance testing and structured training. The primary inclusion criterion was the absence of medical contraindications to performing high-intensity physical training. Exclusion criteria included participation in any additional training programs, recent heat acclimatization, prior training under hypoxic conditions, or passive hypoxic exposure within the previous six months.

Participants were randomly assigned to one of five groups:

1. NOR+21 °C: Training under normoxic conditions (altitude equivalent ~200 m above sea level; FIO_2_ = 20.95%) at a thermoneutral temperature (21 °C);
2. IHT+21 °C: Intermittent hypoxic training at 21 °C (FIO_2_ = 14.4%);
3. NOR+31 °C: Training in heat (31 ± 1 °C) under normoxic conditions;
4. IHT+31 °C: Intermittent hypoxic training in heat conditions (31 ± 1 °C, FIO_2_ = 14.4%);
5. CON: Control group (no training intervention).

The control group was included to provide a reference for changes occurring over the same four-week period without the training intervention, thereby allowing changes observed in the intervention groups to be distinguished from changes related to repeated measurements or the passage of time.

All training protocols were conducted under identical humidity conditions, maintained at 40 ± 1%.

Each group consisted of 16 participants. All training groups completed the same submaximal interval training protocol three times per week for a duration of four weeks. Somatic measurements were taken prior to the training intervention in all groups. Participants were instructed not to alter their dietary habits or physical activity levels during the study period.

The study was approved by the Bioethics Committee of the Regional Medical Chamber in Kraków, Poland (approval no. 47/KB/OIL/2022). The research was conducted in accordance with the ethical standards of the Declaration of Helsinki. Written informed consent was obtained from all participants following a detailed explanation of the study protocol.

### 2.2. Anthropometric Measurements

Anthropometric measurements were conducted prior to the first training session and included assessments of body mass (BM), body composition, and body height (BH). Body mass and composition were measured using an eight-electrode bioelectrical impedance analysis device (Jawon Medical, IOI-353, Seoul, Republic of Korea). All measurements were performed in the morning; participants were adequately hydrated, and their hands and feet were degreased immediately prior to the measurement.

Body composition analysis included fat mass (FM), body fat percentage (%FAT), and lean body mass (LBM). Body mass index (BMI) was calculated for each participant. Body height was measured using a stadiometer (Seca 217, Hamburg, Germany). Body mass was recorded to the nearest 0.1 kg, and body height to the nearest 1 mm.

Body mass was measured immediately before the incremental test and immediately before and after the 1st and 12th training sessions.

### 2.3. Evaluation of Aerobic Capacity, Determination of Workloads and Training

#### 2.3.1. Graded Test

Participants performed the progressive test on a cycle ergometer (eBike, GE HealthCare, Chicago, IL, USA). A ramp protocol was used. After a 4-minute warm-up at 60 watts, the workload was increased by 15 watts per minute until volitional exhaustion. Respiratory indices such as oxygen uptake (VO_2_), carbon dioxide production (VCO_2_), pulmonary ventilation (VE), fractional concentrations of expired CO_2_ (%FECO_2_) and O_2_ (%FEO_2_), and ventilatory equivalents for oxygen (VE/VO_2_) and carbon dioxide (VE/VCO_2_) were measured breath-by-breath using a metabolic gas analyzer (MetaLyzer 3R, Cortex, Leipzig, Germany). Criteria used for determining ventilatory thresholds were as follows: for VT1-minimum FEO_2_ and VE/VO_2_ values; for VT2-maximum FECO_2_, minimum VE/VCO_2_, and a nonlinear increase in VE [33,34]. The aim of the test was to determine aerobic capacity (maximal oxygen uptake VO_2_max) and establish individual training loads corresponding to the first ventilatory threshold (VT1) and the second ventilatory threshold (VT2).

#### 2.3.2. Training

The workload during interval training was individually determined for each participant based on the results of a graded exercise test and corresponded to the intensity of ventilatory thresholds. A constant training load was maintained over the 4-week training period.

The submaximal aerobic interval training was performed on a cycle ergometer (Wattbike, Nottingham, UK) either under hypoxic conditions (FIO_2_ = 14.4%) or in normoxia and lasted 60 min. After a 6-minute warm-up at VT1 power, participants performed 6 sets of 6 min of exercise at VT2 power, interspersed with 3 min of active recovery at VT1 power. At VT1, the highest rate of aerobic metabolism was observed, whereas VT2 represents the greatest exercise intensity at which a balance occurs between lactate production and its clearance during exercise [34]. The first and the last training sessions were monitored, i.e., the physiological response to training was assessed. The loads were selected individually according to the participant’s exercise capacity, and each had the same metabolic background (Table 1).

The NOR+21 °C, IHT+21 °C, NOR+31 °C, and IHT+31 °C groups each completed 12 training sessions (3 sessions per week for 4 weeks), whereas the CON group participated only in two training sessions (baseline and post-intervention), separated by a four-week interval to match the study duration. The participants’ hydration was monitored during the workout. Before the workout (after blood collection), each participant received an isotonic drink (Isodrinx, 700 mL, Nutrend, Olomuniec, Czech Republic), which they were required to consume during the first 30 min of the workout.

Ambient temperature and relative humidity in the hypoxic thermoclimatic chamber were measured using integrated sensors built into the Hypoxico system (Hypoxico, Bickenbach, Germany). Training in hypoxic conditions took place in a thermoclimatic hypoxic chamber (Hypoxico, Bickenbach, Germany), operating using non-nitrogen technology. In this technology, the hypoxia generator prepares hypoxic air with a composition of (O_2_: 14.4%, CO_2_: 0.03%), which is then pumped into the chamber and the air composition in the chamber is monitored to maintain constant conditions.

### 2.4. Physiological Measurements During Training

Physiological parameters were assessed during the first and last interval training sessions. Measurements were taken at 5-minute intervals throughout the exercise sessions. The following physiological indicators were evaluated:

1. Subjective Thermal Sensation
  Assessed using the 7-point Bedford Scale: 1—too cold, 2—cool, 4—comfortable, 5—comfortably warm, 6—too warm, 7—too hot [5].

2. Rating of Perceived Exertion (RPE)
  Assessed using the 20-point Borg Scale: 6–7—extremely light work, 8–9—very light, 10–11—fairly light, 12–13—somewhat hard, 14–15—hard, 16–17—very hard, 18–20—maximal exertion [35,36].

3. Heart Rate (HR)
  Monitored using a Polar 610S heart rate monitor (Polar, Kuopio, Finland).

4. Temperature Measurements
  Tympanic (Ttym) and Skin Temperature (Tsk).

Tympanic temperature (Ttym) was measured using an ear thermistor probe placed in the auditory canal, isolated from external environmental influence. Each participant used the same sensor throughout the study. Data were recorded using a calibrated medical thermometer (CTF 9004; Ellab, Hillerød, Denmark).

Skin temperature (Tsk) was measured using an MHS-A device (Ellab, Hillerød, Denmark), with an accuracy of ±0.05 °C. The weighted mean skin temperature was calculated according to the Hardy and DuBois formula [37,38,39]:

$T_{\bar{sk}}$ = 0.07 forehead + 0.35 abdomen + 0.14 forearm + 0.05 hand + 0.19 anterior thigh + 0.13 anterior calf + 0.07 foot, where all limb temperatures were measured on the right side of the body.

Additionally, prior to the start of both the first and final training sessions, participants remained seated for 30 min and then baseline Tsk and Ttym values were recorded. The analyses utilized data recorded during the final 6-minute exercise bout (at the 55th minute of the training session) post-exercise and pre-exercise values.

### 2.5. Blood Sampling and Laboratory Analyses

During both the first and final training sessions, blood samples were collected from the participants twice while they were seated: before exercise, following 5 min of seated rest, and immediately after completion of the exercise session. To determine lactate concentration [La], 300 µL of blood was collected into microtubes containing EDTA and sodium fluoride as a glycolysis inhibitor. The samples were stored under refrigerated conditions for no longer than 20 min and then centrifuged at 14,300× *g* for 3 min. Immediately after centrifugation, a 10 µL aliquot of plasma was collected, and lactate concentration was determined using the L-Lactate assay (Randox Laboratories Ltd., Crumlin, County Antrim, UK), based on a colorimetric enzymatic method. The analytical sensitivity of the assay was 0.165 mmol/L, and the method was linear up to a concentration of 19.7 mmol/L. Absorbance was measured at 550 nm using an Evolution 201 UV–Vis spectrophotometer (Thermo Fisher Scientific, Waltham, MA, USA). Hemoglobin concentration (Hb) and hematocrit (Hct) were determined in whole blood using a Sysmex XN-1000 automated hematology analyzer (Sysmex Corporation, Kobe, Japan). Hemoglobin concentration was measured using the cyanide-free sodium lauryl sulfate method, whereas erythrocyte parameters, including hematocrit, were determined using electrical impedance with hydrodynamic focusing. Percentage changes in plasma volume (ΔPV) were calculated separately for the first and final training sessions from the corresponding pre- and post-exercise hemoglobin and hematocrit values, using the Dill and Costill equation as modified by Harrison et al. [30,31]. Post-exercise lactate concentrations were adjusted for exercise-induced changes in plasma volume according to the method proposed by Kraemer and Brown [40].

To assess the magnitude of adaptive changes occurring during a single training session (i.e., the first and twelfth sessions), changes in the parameters La, PV, BM, Ttym, and Tskin were expressed as the difference (Δ) between the value recorded after the training session and the value recorded before it began (Δ = post − pre).

### 2.6. Participants

The participants were healthy men who did not engage in regular sports training; however, they did not lead a sedentary lifestyle. They reported irregular, spontaneous physical activity of varying intensity, performed no more than once or twice per week. Physical activity was assessed using the International Physical Activity Questionnaire (IPAQ). Physical activity level was expressed as MET-min/week and calculated according to the standard IPAQ scoring procedure based on the frequency and duration of reported physical activity and the corresponding MET values [41]. The physical activity, fitness, and somatic build of participants in a particular group are shown in Table 2 and Table 3.

### 2.7. Statistical Analysis

Sample size was calculated using G*Power software version 3.1.9.7 (Germany). The following parameters were entered into the program: test family = F tests; statistical test = repeated measures ANOVA, within-between interaction; type of power analysis = a priori: compute required sample size—given α, power, and effect size. Input parameters were as follows: effect size f = 0.25, α error probability = 0.05, power (1–β) = 0.95, number of groups = 5, number of measurements = 2, correlation among repeated measures = 0.5, and nonsphericity correction ε = 1.0. The required total sample size was 80 participants (16 participants per group).

Data are presented as the mean ± standard deviation. Before performing analysis of variance (ANOVA), assumptions of variance were tested: homogeneity of variance was verified using Levene’s test, and data distribution was assessed using the Shapiro–Wilk test. A one-way ANOVA was used to assess differences in physical activity and VO_2_max among the participants. Repeated measures ANOVA was used to detect between-group differences, changes over time (pre- vs. post-intervention), and to assess effect size (partial eta-squared, ηp^2^). The following main effects were analyzed: group, time, and the interaction between these two factors. Effect sizes (ηp^2^) were interpreted as small (0.01), medium (0.06), or large (0.14) [42]. If ANOVA revealed significant effects, Tukey’s post hoc test was applied. Additionally, if post hoc comparisons were significant, Cohen’s d effect size (ES) was calculated for pre- vs. post-intervention comparisons. Effect sizes were interpreted as small (0.20), medium (0.50), or large (0.80) [42].

The data (La, Tym, Tskin, PV, BM) are presented as the difference (Δ) between the highest (post or exercise maximum) and lowest (pre) recorded values.

Statistical analyses were performed using STATISTICA version 13 (StatSoft, Inc., Tulsa, OK, USA). Statistical significance was set at *p* < 0.05.

## 3. Results

We were particularly interested in changes over time in a given variable within a specific group under the combined influence of an environmental stressor and physical exertion. The absolute values of the variables studied and the post-hoc analysis of between-group differences (effect: group) at individual measurement points are presented in the Supplementary Materials (Table S1 and Table S2).

Body mass measurements taken immediately before the graded test are presented in Table 3, while results obtained pre and post the first and twelfth training sessions are shown in Table 4 and Table S1.

No statistically significant differences were observed among the groups in the analyzed somatic parameters or self-reported physical activity levels, except for body mass (*p* = 0.04). Effect sizes prior to the first training session varied depending on the specific parameter measured (Table 3).

When comparing the last training session (after the 4-week intervention) with the first training session within each group, a statistically significant decrease in HR (*p* = 0.04) and in exercise intensity expressed as a percentage of maximal heart rate (*p* = 0.03) was observed in the IHT+21 °C group. Moreover, a significant decrease in perceived exertion was noted in both the IHT+21 °C (*p* = 0.04) and IHT+31 °C (*p* = 0.001) groups. A significant change in tympanic temperature (ΔT_tym_) was also observed only in the IHT+21 °C group, where an increase was recorded (*p* = 0.01). None of the training sessions had a significant effect on plasma volume and lactate concentration or on changes (increases) in these parameters during the training sessions. Similarly, no changes in hemoglobin or hematocrit levels were observed in any group.

Detailed changes in physiological indicators in response to submaximal interval training under combined heat and hypoxia conditions are presented in Table 4 and Table 5.

## 4. Discussion

The aim of the present study was to analyze the impact of training in combined heat and hypoxic conditions on selected physiological responses during submaximal interval training. In addition, we assessed the course of exercise adaptation to training under various environmental conditions. For this purpose, the physiological response recorded during the first and the last training session was compared, treating them as two measurement points. In this study, we did not change the training load (adjusting it to the environmental conditions) because we wanted to isolate the effect of the environment in which the training takes place. Both heat and hypoxia are powerful stressors and they put additional strain on the body during work. The strength of this study lies in the simultaneous combination of two conditions that have the potential to improve submaximal exercise capacity. We were particularly interested in incorporating heat into IHT, which, according to our hypothesis, should elicit a stronger response from the body and subsequently greater adaptive changes during training. The key finding of this study is that, over the course of 4 weeks of training, the physiological response did not change significantly in any group. Only the IHT+21 °C condition resulted in a significant reduction in submaximal HR, which should be considered a beneficial adaptive change. The inclusion of a non-training control group was important for determining whether the observed changes resulted from the environmental training protocols or from time-related effects, repeated testing, and familiarization with the exercise procedures. No significant group × time interactions were observed for Hb, Hct, or ΔPV, indicating that neither heat nor hypoxia, alone or in combination, produced a detectable hematological adaptation beyond that observed in the control condition. Therefore, the significant reduction in heart rate observed in the IHT+21 °C group should be interpreted cautiously, as it represents a within-group change and cannot by itself demonstrate a training effect that differs from the control group. This distinction is important because responses to heat and hypoxia may vary according to training status, exposure duration, hypoxic dose, and the specific combination of environmental stressors.

RPE is a recognized and validated psychophysiological indicator, and its declared level depends on the individual characteristics of the participant and is affected by the mental state of the responders. It should be noted that the values obtained during the first measurement may have been partially influenced by the psychological state of the participants (e.g., stress associated with performing the exercise for the first time). However, the key issue was to analyze whether changes in RPE followed a similar pattern under different environmental conditions. The study recorded a significant increase in subjective perceived exertion (RPE) in all groups training under hypoxic conditions, regardless of ambient temperature. An increase in perceived exertion under hypoxic conditions as well as under combined hypoxia and heat has also been reported by other researchers. Hobbins et al. (2019), in a study involving 19 trained runners, applied an interval running protocol under hypoxia (FiO_2_ 15.0%) and normoxia (FiO_2_ 20.9%) and demonstrated that perceptions of exertion progressively worsened across successive intervals, with the effect being stronger in hypoxia than in normoxia [43]. Similar findings were obtained by Levine et al., who conducted a 2-h walking exercise in 10 volunteers under hypoxia (FiO_2_ 15.0%), heat (38 °C), and the combination of hypoxia and heat (FiO_2_ 15.0% + 38 °C) [44].

In the group exposed to hypoxic training under thermoneutral conditions (IHT+21 °C), a significant decrease in heart rate (HR) and exercise intensity expressed as a percentage of maximal heart rate (%HRmax) was observed. These findings are consistent with previous studies that demonstrated reductions in HRmax and improvements in exercise economy following training under hypoxic conditions [45,46]. Moreover, Maciejczyk et al. [47] reported that hypoxic training in untrained men shifted ventilatory thresholds and increased VO_2_ at VT2, suggesting a lower cardiovascular cost for the same relative exercise intensity. In the same group (IHT+21 °C), an increase in tympanic temperature (ΔTtym) was also observed. Previous studies indicate that hypoxic conditions may influence the dynamics of heat transfer in the body, including tympanic temperature [48], and that Ttym is associated with cardiovascular strain during exercise [49]. Our findings therefore suggest an increase in metabolism and greater load on thermoregulatory mechanisms in response to training under hypoxic conditions.

Hypoxia and physical exercise are independent yet potent metabolic stressors [50,51]. Consequently, exercise under hypoxia significantly decreases mitochondrial oxygen partial pressure, simultaneously reducing oxygen delivery and increasing its demand [52]. The observed decrease in arterial pressure results from hypoxia-induced vasodilation [53]. The significant reductions in HR and %HRmax in the IHT+21 °C group between the first and twelfth training session may indicate improved cardiorespiratory adaptation to hypoxic training. These findings are consistent with Millet et al. [54], who demonstrated that hypoxic exposure enhances blood flow distribution, improving muscle efficiency. This effect is attributed to enhanced angiogenesis, increased muscle capillarization, and this improved oxidative capacity of muscle fibers. Hypoxia also stimulates the expression of transcription factors such as hypoxia-inducible factor 1-alpha (HIF-1α), which increases erythropoietin production and consequently improves oxygen transport in the blood. This leads to enhanced oxygen delivery to active muscles, delayed onset of fatigue, and improved endurance performance [54]. Cardiovascular responses to hypoxic training, such as reduced HR, result from modifications in cardiac function that help protect the body from excessive load [22]. It should be emphasized that HR, although widely used in monitoring training loads, is not free from limitations, as it may be modulated by factors such as body temperature. In the present analysis, however, it was considered as one of several evaluated indicators. The key aspect was the change in HR values between the first and the last training session within the same group. This approach made it possible to assess the influence of environmental conditions on the physiological response.

Coombs et al. [55] discussed the influence of hypoxic training on thermoregulatory mechanisms and showed that regular hypoxic exposure leads to cardiovascular and respiratory adaptations that may influence thermoregulatory responses. In the present study, a significant increase in tympanic temperature (ΔTtym) was observed between the first and last training sessions in the normobaric hypoxia group (IHT+21 °C), despite minimal changes in skin temperature. This may reflect increased cellular metabolism and altered blood flow distribution due to reduced oxygen availability. Such adaptation may lead to greater internal heat production and changes in the efficiency of cooling mechanisms such as sweating and vasodilation. Interestingly, we also did not observe an increase in plasma volume.

The increase in RPE in the IHT+21 °C group may reflect a combination of central and metabolic adaptive mechanisms. Although physiological indicators such as HR and %HRmax decreased, suggesting improved cardiac efficiency, the concurrent increase in RPE indicates elevated subjective strain. One possible explanation is increased activity of group III and IV afferent nerve fibers, which transmit fatigue-related signals to the central nervous system [56]. Their activity may be intensified under hypoxia due to local acidosis and hypoxemia, resulting in enhanced signaling to the somatosensory cortex and regions involved in processing fatigue-related stimuli. Additionally, hypoxia may influence central neurotransmitter balance—particularly serotonin and dopamine—leading to earlier activation of inhibitory mechanisms that reduce the motivation to continue exercise [57]. According to the “central governor” theory, exercise under hypoxia may be perceived by the brain as a threat to homeostasis earlier than under normoxic conditions, even when objective physiological parameters remain within normal limits [58,59]. The increase in RPE may also result from perceptual adaptation—hypoxia alters how the body interprets effort-related stimuli. This has been observed in athletes acclimatized to high altitudes, where discrepancies between physiological load and perceived exertion were reported [60].

Due to logistical and technical constraints, such as the difficulty of simultaneous exposure to heat and hypoxia, these stressors have traditionally been applied sequentially rather than concurrently [59,60]. Combining them within a single training protocol may offer a more effective environmental strategy to enhance aerobic performance [61]. Notably, in the present study, no significant changes in physiological parameters were found in the IHT+31 °C group, except for a significant increase in perceived exertion (RPE). This may be attributed to excessively heat ambient temperature, which may reduce skin blood flow and muscle perfusion under hypoxic conditions, thereby limiting oxygen delivery to active muscle tissue without clear changes in classical physiological parameters such as heart rate or ventilation [62].

Moreover, the simultaneous presence of environmental heat stress and physical exertion intensifies physiological strain, manifesting in increased core, skin, and brain temperatures; augmented cardiovascular responses; greater reliance on carbohydrate metabolism; and decreased aerobic performance [22,53]. Under combined environmental stress, the central nervous system may prematurely activate protective mechanisms aimed at limiting further physiological burden, resulting in an increased subjective sense of exertion. Hypoxia may further modulate neurotransmission, contributing to elevated fatigue perception [11]. It has also been shown that heat combined with high humidity impairs sweat evaporation efficiency, leading to heat accumulation in the body and elevated RPE values [61]. Similar findings were reported by Périard et al. [22], who demonstrated that elevated core temperature impairs thermal comfort and increases perceived exertion. Furthermore, Périard et al. [26] found that cumulative exposure to heat and humidity reduces sweat evaporation efficiency, thereby limiting the body’s thermoregulatory capacity.

Hematological responses and applicability of the findings. Contrary to the expected heat-related haemodilution and hypoxia-related hemoconcentration, no significant pre-to-post changes in hemoglobin concentration (Hb) or hematocrit (Hct) were observed in any group. Mean Hb values remained approximately within the range of 14.7–15.6 g/dL, whereas Hct values remained approximately within the range of 43–46%, indicating no systematic shift in either hematological marker during the intervention. These findings suggest that four weeks of submaximal interval training performed three times per week under normoxic or normobaric hypoxic conditions, with or without additional heat exposure, did not induce a detectable persistent hematological adaptation in healthy young men who were not regularly engaged in structured sports training. The participants were aged 19–26 years and had no recent exposure to heat or hypoxic acclimatization, whereas hypoxic training was performed at an inspired oxygen fraction of 14.4% and heat exposure at 31 ± 1 °C. The absence of significant changes in exercise-induced plasma-volume changes (ΔPV) between the first and final training sessions further indicates that the applied training duration and environmental dose were insufficient to produce a consistent alteration in plasma-volume regulation. The ΔPV values remained close to zero and showed considerable interindividual variability across the experimental groups. Importantly, the significant reduction in heart rate observed in the IHT+21 °C group should not be attributed to measurable plasma-volume expansion or changes in Hb and Hct, because neither these hematological markers nor ΔPV changed significantly after the intervention. This finding suggests that the observed reduction in cardiovascular strain may have resulted from non-hematological adaptations, such as improved cardiovascular efficiency, altered peripheral blood-flow regulation, or improved exercise tolerance at the same relative workload.

The present findings are therefore primarily applicable to healthy young men with low or moderate habitual physical activity, limited training experience, and no recent heat or hypoxic acclimatization. They should not be directly extrapolated to endurance-trained athletes, women, older adults, clinical populations, or individuals exposed to longer, more severe, or continuous heat and hypoxic stimuli. The lack of changes in Hb and Hct should also be interpreted as an absence of a detectable systematic response under the present experimental conditions, rather than as evidence that no transient fluid shifts occurred. Heat acclimation may induce plasma-volume expansion within the first days of exposure, whereas prolonged hypoxic exposure may additionally stimulate erythropoietic adaptations [26,29].

Finally, Hb and Hct are concentration-based variables, and ΔPV was estimated indirectly from pre- and post-exercise Hb and Hct values using the Dill and Costill equation, as modified by Harrison et al. [30,31]. Therefore, small or short-lasting changes in plasma volume may not have been detected, particularly given the potential influence of hydration status, sampling timing, and measurement variability.

### Study Limitations

A limitation of the present study is the relatively short duration of the intervention, which may not fully reflect the long-term physiological adaptations to combined environmental stressors. In the present study, neither the level of physical fitness nor the mechanisms underlying the observed differences were analyzed.

In addition, only untrained men participated in the study, which may represent a significant limitation. Sex-related differences in thermoregulation—including the influence of female sex hormones, differences in body surface-to-mass ratio, fat distribution, as well as variations in sweating mechanisms and cutaneous blood flow—may affect the physiological response to exercise under hypoxic and heat conditions [63]. Therefore, the present findings should not be directly generalized to female populations or trained men. Future research should address these limitations by extending the duration of the intervention and conducting analogous experiments in women, which would allow for a more comprehensive assessment of physiological responses under combined environmental stressors.

The loads during training were individualized and selected according to ventilatory thresholds (i.e., exercise capacity), however, in the graded test, the loads were not individualized, e.g., according to body mass. Previous studies [47,64,65] indicated that intermittent hypoxic training does not induce erythropoietin (EPO) concentration changes, therefore we decided not to examine EPO in this study. This was partially confirmed by the results of our study, in which no significant changes in Hct and Hb were observed as a result of training.

In this study, a low rate of power increase (15 W/min) was used in the ramp protocol. Buchfuhrer et al. [66] compared cycle tests of various changes in work rates and noted that ramping at an intermediate rate (30 W/min) may produce greater VO_2_max values; however, this study did not evaluate the effect of training on VO_2_max.

Skin temperature measurement should be performed on the left side of the body.

## 5. Conclusions

The results are ambiguous, and it is likely that the adaptation (training) period or the frequency and duration of training were insufficient to observe full adaptation, although adaptive trends were evident. The four-week interval training program under normobaric hypoxia and thermoneutral conditions (IHT+21 °C) resulted in a significant reduction in heart rate during exercise and in exercise, compared to the first training session, indicating improved exercise tolerance. The combination of hypoxic and heat training did not yield the expected results, and no significant changes in the physiological response to submaximal aerobic training were observed in this group.

## Figures and Tables

**Table 1:** Characteristics of the training loads.

Variable	Condition	Mean ± SD	ANOVA f *p* ƞp^2^
VT1_P	CON	82.5 ± 17.0	2.4 0.06 0.11
	NOR+21 °C	70.8 ± 17.6	
	NOR+31 °C	74.9 ± 16.5	
	IHT+21 °C	90.5 ± 22.8	
	IHT+31 °C	81.7 ± 23.8	
VT1_%Pmax	CON	30.3 ± 5.6	3.4 0.01 0.15
	NOR+21 °C	24.9 ± 5.9	
	NOR+31 °C	28.2 ± 4.1	
	IHT+21 °C	31.8 ± 7.2	
	IHT+31 °C	27.8 ± 5.2	
VT1_%HRmax	CON	66.8 ± 6.3	1.03 0.39 0.05
	NOR+21 °C	63.3 ± 5.1	
	NOR+31 °C	64.6 ± 4.2	
	IHT+21 °C	64.5 ± 4.8	
	IHT+31 °C	64.1 ± 5.3	
VT1_%VO_2_max	CON	38.1 ± 4.4	3.26 0.01 0.14
	NOR+21 °C	34.7 ± 4.9	
	NOR+31 °C	40.4 ± 5.0	
	IHT+21 °C	39.3 ± 5.8	
	IHT+31 °C	40.1 ± 5.2	
VT2_P	CON	166.2 ± 20.2	4.26 0.003 0.18
	NOR+21 °C	162 ± 21.1	
	NOR+31 °C	145.5 ± 30.4	
	IHT+21 °C	183.8 ± 29.2	
	IHT+31 °C	164.1 ± 30.7	
VT2_%Pmax	CON	61.4 ± 7.9	4.94 0.001 0.20
	NOR+21 °C	57.4 ± 8.6	
	NOR+31 °C	54.7 ± 6.6	
	IHT+21 °C	64.5 ± 6.8	
	IHT+31 °C	56.4 ± 5.8	
VT2_%HRmax	CON	83.4 ± 6.3	2.48 0.05 0.11
	NOR+21 °C	80.5 ± 6.0	
	NOR+31 °C	78.7 ± 5.7	
	IHT+21 °C	83.4 ± 3.8	
	IHT+31 °C	80.5 ± 4.4	
VT2_%VO_2_max	CON	64.1 ± 8.1	1.09 0.36 0.05
	NOR+21 °C	61.2 ± 9.8	
	NOR+31 °C	62.4 ± 7.6	
	IHT+21 °C	66.4 ± 5.8	
	IHT+31 °C	63.6 ± 5	

VT1—first ventilatory threshold; VT2—second ventilatory threshold; HRmax—maximal heart rate; P—power; VO_2_max—maximal oxygen uptake; NOR+21—training in thermoneutral and normoxia; IHT+21—training in thermoneutral and hypoxia; NOR+31—training in heat and normoxia, IHT+31—training in heat and hypoxia; CON-control group.

**Table 2:** Physical activity and maximal oxygen uptake (VO_2_max) of the participants before training.

Variable	Condition	Mean ± SD	ANOVA f *p* ƞp^2^
METs-min/week	CON	6582 ± 2831	1.96 0.10 0.09
	NOR+21 °C	6154 ± 2509	
	NOR+31 °C	4936 ± 2068	
	IHT+21 °C	6376 ± 1881	
	IHT+31 °C	6944 ± 1580	
VO_2_max (L/min)	CON	3.34 ± 0.43	3.9 0.005 0.17
	NOR+21 °C	3.55 ± 0.37	
	NOR+31 °C	3.59 ± 0.53	
	IHT+21 °C	3.41 ± 0.55	
	IHT+31 °C	3.02 ± 0.43	

MET—metabolic equivalent of task; VO_2_max—maximal oxygen uptake.

**Table 3:** Age and body build of the participants before first training.

Variable	Condition	Mean ± SD	ANOVA f *p* ƞp^2^
Age (yrs)	CON	22.88 ± 2.91	2.47 0.05 0.11
	NOR+21 °C	20.50 ± 1.03	
	NOR+31 °C	22.47 ± 3.67	
	IHT+21 °C	21.50 ± 1.55	
	IHT+31 °C	22.13 ± 1.57	
BH (cm)	CON	178.9 ± 5.9	1.61 0.18 0.07
	NOR+21 °C	179.7 ± 5.6	
	NOR+31 °C	177.0 ± 5.8	
	IHT+21 °C	182.0 ± 5.5	
	IHT+31 °C	179.1 ± 5.7	
BM (kg)	CON	75.8 ± 11.5	0.63 0.04 0.03
	NOR+21 °C	76.7 ± 8.3	
	NOR+31 °C	74.2 ± 12.1	
	IHT+21 °C	79.6 ± 10.4	
	IHT+31 °C	76.8 ± 7.3	
BF (%)	CON	18.3 ± 5.3	0.18 0.94 0.009
	NOR+21 °C	17.6 ± 3.9	
	NOR+31 °C	18.4 ± 6.8	
	IHT+21 °C	17.4 ± 3.9	
	IHT+31 °C	18.5 ± 3.4	
BMI (kg/m^2^)	CON	23.6 ± 2.9	0.11 0.97 0.005
	NOR+21 °C	23.7 ± 2.2	
	NOR+31 °C	23.5 ± 3.2	
	IHT+21 °C	24.0 ± 2.3	
	IHT+31 °C	23.9 ± 1.6	

BH—body height; BM—body mass; BF—body fat; BMI—body mass index; NOR+21—training in thermoneutral and normoxia; IHT+21—training in thermoneutral and hypoxia; NOR+31—training in heat and normoxia, IHT+31—training in heat and hypoxia; CON—control group.

**Table 4:** Changes in physiological responses to submaximal interval exercise during the first and final training sessions under different environmental conditions.

Variable	Condition	Pre	Post		ANOVA		Post-Hoc	Post-Hoc
				Effect: Group F *p* ƞp^2^	Effect: Time F *p* ƞp^2^	Interaction F *p* ƞp^2^	Pre vs. Post *p*	d-Cohen
HR (bpm)	CON	154.5 ± 16.2	149.47 ± 19.44	5.34 <0.001 0.21	13.02 <0.001 0.14	1.18 0.32 0.05	0.94	0.28
	NOR+21 °C	154.44 ± 16.74	146.69 ± 15.0				0.60	0.49
	NOR+31 °C	167.65 ± 13.76	164.18 ± 9.48				0.99	0.30
	IHT+21 °C	161.63 ± 14.9	148.94 ± 15.17				0.04	0.84
	IHT+31 °C	166.69 ± 18.5	164.63 ± 8.85				0.99	0.15
%HRmax	CON	84.46 ± 8.71	81.62 ± 9.21	3.91 0.006 0.17	13.30 <0.001 0.14	1.30 0.27 0.06	0.92	0.32
	NOR+21 °C	84.02 ± 9.24	79.74 ± 7.8				0.57	0.50
	NOR+31 °C	89.66 ± 5.39	87.94 ± 5.12				0.99	0.33
	IHT+21 °C	89.21 ± 7.65	82.17 ± 7.61				0.03	0.92
	IHT+31 °C	89.2 ± 9.6	88.16 ± 5.34				0.99	0.14
ΔBM (kg)	CON	−0.02 ± 0.34	−0.05 ± 0.21	8.32 <0.001 0.30	3.05 0.08 0.03	0.15 0.96 0.007	1.00	-
	NOR+21 °C	0.02 ± 0.22	−0.05 ± 0.15				0.99	-
	NOR+31 °C	0.40 ± 0.82	0.27 ± 0.32				0.97	-
	IHT+21 °C	0.26 ± 0.33	0.12 ± 0.23				0.97	-
	IHT+31 °C	0.49 ± 0.43	0.40 ± 0.32				0.99	-
ΔT_tym_ (°C)	CON	0.74 ± 0.55	0.81 ± 0.62	14.13 <0.001 0.42	3.61 0.06 0.04	3.10 0.02 0.14	0.99	0.12
	NOR+21 °C	0.63 ± 0.52	0.68 ± 0.68				1.00	0.08
	NOR+31 °C	1.38 ± 0.43	1.52 ± 0.61				0.99	0.27
	IHT+21 °C	0.23 ± 0.49	0.79 ± 0.60				0.01	1.14
	IHT+31 °C	1.56 ± 0.67	1.39 ± 0.54				0.97	0.28
ΔTskin (°C)	CON	−0.24 ± 1.1	−0.03 ± 0.7	1.24 0.29 0.06	1.13 0.28 0.01	1.10 0.35 0.05	-	-
	NOR+21 °C	0.04 ± 0.54	−0.46 ± 0.09				-	-
	NOR+31 °C	0.08 ± 0.59	0.03 ± 0.82				-	-
	IHT+21 °C	−0.08 ± 0.46	−0.24 ± 0.51				-	-
	IHT+31 °C	0.18 ± 0.36	0.12 ± 1.06				-	-
ΔLa (mmol/L)	CON	2.42 ± 1.60	2.14 ± 1.79	4.49 0.34 0.05	7.01 0.04 0.05	4.84 0.02 0.13	0.99	0.18
	NOR+21 °C	2.64 ± 2.71	1.27 ± 0.89				0.05	0.76
	NOR+31 °C	2.18 ± 0.84	2.72 ± 1.22				0.96	0.64
	IHT+21 °C	3.47 ± 2.10	2.56 ± 1.44				0.56	0.51
	IHT+31 °C	2.43 ± 1.58	2.44 ± 1.50				1.00	0.01
ΔPV (%)	CON	0.77 ± 5.13	1.69 ± 4.77	0.13 0.21 0.07	2.33 0.13 0.02	0.75 0.55 0.33	-	-
	NOR+21 °C	0.35 ± 5.11	1.28 ± 3.52				-	-
	NOR+31 °C	−0.97 ± 4.12	−0.77 ± 3.23				-	-
	IHT+21 °C	−2.15 ± 3.72	−1.12 ± 4.29				-	-
	IHT+31 °C	−0.09 ± 4.59	−0.60 ± 3.43				-	-
Bedford scale (AU)	CON	4.82 ± 1.07	4.88 ± 1.05	3.39 0.01 0.14	8.48 0.004 0.09	1.61 0.18 0.07	1.00	0.06
	NOR+21 °C	4.75 ± 1.13	4.63 ± 0.72				0.99	0.13
	NOR+31 °C	5.82 ± 0.73	5.24 ± 1.09				0.98	0.79
	IHT+21 °C	5.19 ± 1.05	4.94 ± 0.85				0.25	0.26
	IHT+31 °C	5.88 ± 0.96	5.25 ± 1.12				0.22	0.61
RPE [Borg Scale] (AU)	CON	12 ± 2.06	11.76 ± 1.79	3.70 0.008 0.16	24.20 <0.001 0.24	3.11 0.02 0.13	0.99	0.12
	NOR+21 °C	13.56 ± 2.45	11.69 ± 1.78				0.04	0.88
	NOR+31 °C	13.59 ± 2.03	13.24 ± 2.02				0.99	0.17
	IHT+21 °C	12.75 ± 1.88	11.63 ± 2.63				0.59	0.60
	IHT+31 °C	15.31 ± 2.5	12.81 ± 2.61				0.001	0.98

HR—heart rate; %HRmax—percentage of maximal heart rate; ΔBM—change in body weight; ΔTtym (°C)—change in tympanic temperature; ΔTskin—change in skin temperature; ΔLa—exercise-induced change in blood lactate concentration; ΔPV—change in plasma volume; RPE—Borg scale; NOR+21—training in thermoneutral and normoxia; IHT+21—training in thermoneutral and hypoxia; NOR+31—training in heat and normoxia; IHT+31—training in heat and hypoxia; CON—control group; (Δ = post-exercise − pre-exercise).

**Table 5:** Changes in biochemical indicators under the influence of combined training in heat and hypoxia during submaximal interval training.

Variable	Condition	1st Training		12st Training		1st Training Post-Hoc	12st Training Post-Hoc	Post-Hoc
		Pre	Post	Pre	Post	Pre vs. Post *p*	Pre vs. Post *p*	1st Pre vs. 12st Pre *p*
Hb (g/dL)	CON	15.40 ± 1.04	15.44 ± 0.97	15.23 ± 1.04	15.14 ± 1.08	NS	NS	NS
	NOR+21 °C	15.54 ± 0.91	15.60 ± 0.74	15.25 ± 1.04	15.16 ± 0.91	NS	NS	NS
	NOR+31 °C	14.82 ± 1.18	14.98 ± 1.35	14.73 ± 1.03	14.84 ± 0.94	NS	NS	NS
	IHT+21 °C	15.11 ± 0.92	15.38 ± 0.86	14.89 ± 0.89	15.00 ± 0.88	NS	NS	NS
	IHT+31 °C	15.35 ± 0.84	15.52 ± 0.93	15.15 ± 1.13	15.38 ± 1.12	NS	NS	NS
Hct (%)	CON	45.3 ± 3.0	44.6 ± 2.5	44.7 ± 2.9	44.1 ± 2.4	NS	NS	NS
	NOR+21 °C	45.6 ± 2.6	45.2 ± 2.1	44.9 ± 3.2	43.9 ± 2.6	NS	NS	NS
	NOR+31 °C	43.3 ± 2.5	43.3 ± 3.0	43.4 ± 2.3	43.4 ± 2.0	NS	NS	NS
	IHT+21 °C	43.3 ± 2.9	43.7 ± 2.4	43.7 ± 2.7	44.0 ± 2.9	NS	NS	NS
	IHT+31 °C	44.9 ± 2.6	44.3 ± 2.5	44.4 ± 3.4	44.7 ± 3.2	NS	NS	NS

Hb—hemoglobin concentration; Hct—hematocrit; NOR+21—training in thermoneutral and normoxia; IHT+21—training in thermoneutral and hypoxia; NOR+31—training in heat and normoxia; IHT+31—training in heat and hypoxia; CON—control group; NS—non-significant.

## Data Availability

Data are contained within the article or Supplementary Materials.

## Supplementary Materials

The following supporting information can be downloaded at https://www.mdpi.com/article/10.3390/jfmk11030344/s1, Table S1: Changes in parameters in response to combined training under high-temperature and hypoxic conditions during submaximal interval training; Table S2: Results of the post-hoc analysis: between-group differences in the first training session (pre) and the 12th training session (post). The analysis was performed only for variables in which a significant main effect (group) was observed in the ANOVA (Table 4).
