# Supplementary material for: Effects of Combined Heat and Hypoxic Training on the Physiological Responses During Submaximal Interval Exercise in Untrained Men
Source: J Funct Morphol Kinesiol. 2026 Aug 31;11(3):344. doi: 10.3390/jfmk11030344 (PMC13608514; doi:10.3390/jfmk11030344)
Supplement: Supplementary file 1 [file jfmk-11-00344-s001.zip › jfmk-4505731-supplementary.pdf]

Table S1. Changes in parameters in response to combined training under high-temperature and hypoxic conditions during submaximal interval training

Variable	Condition	1st training		12st training		1st training post-hoc	12st training post-hoc
		Pre	Post	Pre	Post	Pre vs. Post p	Pre vs. Post p
BM (kg)	CON	76.2±11.76	76.22±11.54	76.23±11.97	76.29±11.95	1.0	0.99
	NOR+21°C	76.97±8.32	76.95±8.31	77.20±8.62	77.25±8.58	1.0	0.99
	NOR+31°C	74.65±12.22	74.24±12.21	74.27±12.33	74.00±12.27	0.02	0.001
	IHT+21°C	79.83±10.18	79.57±10.01	79.64±9.72	79.52±9.82	0.49	0.68
	IHT+31°C	76.99±7.10	76.50±6.82	76.89±6.77	76.49±6.69	0.003	<0.001
Ttym (°C)	CON	36.16±0.41	36.90±0.44	35.97±0.70	36.79±0.34	<0.001	<0.001
	NOR+21°C	36.11±0.54	36.75±0.42	35.75±0.51	36.43±0.50	<0.001	0.001
	NOR+31°C	36.37±0.36	37.75±0.50	35.88±0.55	37.40±0.23	<0.001	<0.001
	IHT+21°C	36.33±0.34	36.56±0.41	35.87±0.52	36.66±0.37	0.77	<0.001
	IHT+31°C	36.01±0.42	37.57±0.50	36.03±0.38	37.42±0.32	<0.001	<0.001
Tskin (°C)	CON	33.88±0.16	33.65±1.04	33.53±0.57	33.49±0.31	0.89	1.00
	NOR+21°C	33.63±0.51	33.67±0.34	28.84±0.60	28.38±0.60	1.00	0.42
	NOR+31°C	33.89±0.49	33.98±0.36	33.39±0.44	33.41±0.72	0.99	1.00
	IHT+21°C	33.90±0.14	33.83±0.37	33.64±0.29	33.41±0.47	0.99	0.97
	IHT+31°C	33.51±0.47	33.70±0.45	29.02±0.84	29.15±0.77	0.98	0.99
La (mmol/L)	CON	1.44±0.39	3.87±1.62	1.42±0.42	3.56±1.70	<0.001	<0.001
	NOR+21°C	1.78±0.53	4.49±2.71	1.71±0.39	2.98±1.00	<0.001	0.01
	NOR+31°C	1.64±0.43	3.82±1.03	1.50±0.34	4.22±1.22	<0.001	<0.001
	IHT+21°C	1.38±0.42	4.85±1.95	1.57±0.43	4.13±1.46	<0.001	<0.001
	IHT+31°C	1.63±0.44	4.07±1.55	1.68±0.41	4.12±1.39	<0.001	<0.001

BM- body mass; **Ttym (°C)**- tympanic temperature; **Tskin**- skin temperature; **La** –lactate concentration; **NOR+21°C**-training in thermoneutral and normoxia; **IHT+21°C**-training in thermoneutral and hypoxia; **NOR+31°C**-training in heat and normoxia, **IHT+31°C**-training in heat and hypoxia; **CON**-control group;

Table S2. Results of the post-hoc analysis: between-group differences in the first training session (pre) and the 12th training session (post). The analysis was performed only for variables in which a significant main effect (group) was observed in the ANOVA (table 4).

Comparison	measurement	Post hoc: intergroup differences p					
		HR (bpm)	%HRmax	ΔTtym (°C)	Bedford Scale (AU)	RPE (AU)	ΔBM (kg)
CON vs. NOR+21°C	pre	1.00	1.00	1.00	1.00	0.57	1.00
	post	0.99	1.00	1.00	0.99	1.00	1.00
CON vs. NOR+31°C	pre	0.25	0.63	0.04	0.10	0.52	0.04
	post	0.13	0.34	0.01	0.98	0.63	0.30
CON vs. IHT+21°C	pre	0.94	0.76	0.25	0.98	0.99	0.52
	post	1.00	1.00	1.00	1.00	1.00	0.95
CON vs. IHT+31°C	pre	0.38	0.76	0.001	0.07	< 0.001	0.01
	post	0.11	0.31	0.12	0.98	0.94	0.02
NOR+21°C vs. NOR+31°C	pre	0.27	0.53	0.01	0.06	1.00	0.12
	post	0.03	0.07	0.001	0.75	0.58	0.35
NOR+21°C vs.IHT+21°C	pre	0.94	0.67	0.60	0.96	0.99	0.77
	post	1.00	1.00	1.00	0.99	1.00	0.96
NOR+21°C vs.IHT+31°C	pre	0.40	0.67	< 0.001	0.05	0.42	0.02
	post	0.03	0.06	0.02	0.74	0.91	0.03
NOR+31°C vs. IHT+21°C	pre	0.98	1.00	< 0.001	0.70	0.99	0.99
	post	0.11	0.50	0.01	1.00	0.52	0.98
NOR+31°C vs. IHT+31°C	pre	1.00	1.00	1.00	1.00	0.42	1.00
	post	1.00	1.00	1.00	1.00	1.00	0.99
IHT+21°C vs. IHT+31°C	pre	0.99	1.00	< 0.001	0.62	0.03	0.79
	post	0.10	0.50	0.10	0.99	0.88	0.53

HR- heart rate; **%HRmax**- percentage of maximal heart rate; **ΔBM**- change in body weight; **ΔTtym (°C)**-change in tympanic temperature; **ΔPV-** change in plasma volume**;** **RPE**- Borg scale; **NOR+21°C**-training in thermoneutral and normoxia; **IHT+21°C**-training in thermoneutral and hypoxia; **NOR+31°C**-training in heat and normoxia, **IHT+31°C**-training in heat and hypoxia; **CON**-control group; (Δ = post-exercise − pre-exercise)
